# Natural Polymers as Carriers for Encapsulation of Volatile Oils: Applications and Perspectives in Food Products

**DOI:** 10.3390/polym16081026

**Published:** 2024-04-09

**Authors:** Ovidiu Tița, Maria Adelina Constantinescu, Lăcrămioara Rusu, Mihaela Adriana Tița

**Affiliations:** 1 Department of Agricultural Sciences and Food Engineering, Lucian Blaga University of Sibiu, Doctor Ion Rațiu No. 7, 550012 Sibiu, Romania; ovidiu.tita@ulbsibiu.ro (O.T.); mihaela.tita@ulbsibiu.ro (M.A.T.); 2 Department of Chemical Engineering and Food, Vasile Alecsandri University of Bacău, 600115 Bacău, Romania

**Keywords:** natural polymers, encapsulation, dairy products, volatile oils, bioactive compounds

## Abstract

The technique of encapsulating different materials into matrices that can both protect and release their contents under specific circumstances is known as encapsulation. It serves the primary function of shielding delicate components from outside influences, including heat, light, and humidity. This can be accomplished by a variety of procedures that, depending on the method and materials selected, result in the creation of particles with various structures. The materials used for encapsulation in food applications must be of high quality, acceptable for human consumption, and stable during processing and storage. The most suitable natural polymers for food applications are carbohydrates, proteins, or mixtures thereof. Volatile oils are end products of plant metabolism, accumulated and stored in various plant organs, cells, or secretory tissues. These are natural and are characterized by the scent of the aromatic plants they come from. Because of their antibacterial and antioxidant qualities, they are being utilized more and more in the food and pharmaceutical industries. Since volatile oils are highly sensitive to environmental changes, they must be stored under specific conditions after being extracted from a variety of plant sources. A promising method for increasing the applicability of volatile oils is their encapsulation into colloidal particles by natural polymers such as carbohydrates and proteins. Encapsulation hides the unfavorable taste of nutrients while shielding delicate dietary ingredients from the effects of heat, moisture, oxygen, and pH. This technique results in improved stability for volatile oils that are often sensitive to environmental factors and offers the possibility of using them in an aqueous system even if they are insoluble in water. This paper aims to provide an overview of the current advances in volatile oil encapsulation technologies and presents a variety of natural polymers used in the food industry for encapsulation. Also, a distinct section is created to highlight the current advances in dairy products enriched with encapsulated volatile oils.

## 1. Introduction

Volatile oils are aromatic volatile liquids that come from various plant sections and are made up of many hydrophobic components [1]. Numerous studies have demonstrated the significant health benefits that consumers receive from volatile oils derived from a variety of plants. These oils are taken from the plant’s root system or its aerial portion [2]. Volatile oils are produced by aromatic and therapeutic plants as secondary metabolites. These oils have a wide range of uses in the culinary, cosmetics, and fragrance industries. Because essential oils contain several active ingredients (e.g., terpenes, terpenoids, carotenoids, coumarins, and curcumins) that are highly significant to the food business, particularly the dairy industry, they have strong antibacterial and food preservation qualities [3]. Because of their antibacterial and antioxidant qualities, volatile oils are being employed more and more in the food and pharmaceutical industries. Finding new uses for natural chemicals has become more of a priority due to the growing interest in these molecules [4]. The potential to use natural, safe, economical, renewable, and easily biodegradable antimicrobial chemicals for short-term food preservation is thus made possible by the diverse qualities of essential oils [3].

Various extraction methods are used to obtain the volatile oils, and then these are analyzed using gas chromatography coupled with mass spectrometry (GC-MS) [5]. Conventional extraction methods are hydro distillation (HD) and steam distillation (SD), which are commonly used. Several innovative green extraction methods have recently been developed to extract essential oil, such as microwave-assisted extraction (MAE), supercritical fluid extraction (SFE) [6], and ultrasound-assisted extraction (UAE) [7].

Because of an imbalance between reactive oxygen species (ROS) and the antioxidant defense system, free radicals can cause oxidative stress, which can lead to several diseases, including hypertension, cancer, cardiovascular disease, neurodegenerative diseases, rheumatoid arthritis, atherosclerosis, and acquired immunodeficiency syndrome (AIDS) [8]. ‘Oxidative stress’ occurs when the body’s antioxidant system is unable to keep up with the generation of free radicals. To attain a balance between antioxidants and free radicals, an external source of antioxidants is required [2]. For detoxification, plants create a variety of enzymatic and non-enzymatic antioxidant systems. Particularly abundant in phenolic chemicals that are antioxidants are herbs. Their redox characteristics and chemical makeup, which are crucial in scavenging free radicals and peroxides, are the sources of their antioxidant action [9]. The main chemical compounds of some essential oils that provide antioxidant activity are shown in Table 1.

Eating food tainted with germs, viruses, parasites, or chemicals like heavy metals can result in over 200 ailments. Foodborne illnesses can arise from food contamination at any point in the chain from food manufacturing to delivery to consumption. These types of diseases include a broad spectrum of disorders, ranging from cancer to diarrhea. Though they can also cause neurological, gynecological, and immunological symptoms, the majority manifest as gastrointestinal problems. Diarrheal diseases are a serious global health concern, with low- and middle-income nations and children under the age of five bearing a disproportionate share of the burden [12].

Unsafe food puts billions of people worldwide at risk. Every year, eating contaminated food causes hundreds of thousands of illnesses and deaths [13]. In the food industry, the quality and safety of prepared or processed food is of a premium importance. Microorganisms present in food can lead to the deterioration of food product quality and, if ingested by humans, can cause infection and disease [14]. Every year, 420,000 people die from foodborne illnesses and 600 million cases of foodborne illness are brought on by unfit food. According to these statistics, foodborne illnesses account for 7.69% (600 million) of the world’s population (7.8 billion) and cause 420,000 fatalities yearly, or 7.5% of all deaths [15]. Both developed and underdeveloped nations worry about bacterial food illnesses. The two main causes of foodborne illness in Europe are *Campylobacter* and *Salmonella*. Apart from campylobacteriosis, which had 246,571 documented cases, the European Centre for Disease Prevention and Control (ECDC) claims that *Salmonella* is the pathogen that caused the greatest number of human infections in the EU in 2018, infecting 91,857 individuals [16]. The majority of foodborne outbreaks connected to retail food operations are caused by food workers contaminating ready-to-eat items. Food workers’ avoidable behaviors are directly linked to four of the five risk factors for foodborne illness associated with food establishments: improper holding, time, and temperature; insufficient cooking temperatures; contaminated equipment and protection from contamination; and poor personal hygiene [17]. Food rotting, the production of toxins, and a decline in food quality can all be caused by the presence and proliferation of microbes [18]. Eating contaminated food causes several illnesses and is a global public health concern [19]. Foodborne illnesses need to be mitigated and prevented because they afflict a large number of people worldwide. Research on plant applications has paved the way for the creation of novel medications and food products, as well as for the discovery of hitherto undiscovered plant qualities [20].

Food shelf life and consumer acceptance both depend on food preservation [21]. Increasingly, food manufacturers are required to extend the shelf life of their products and maintain their appearance for as long as possible. Natural preservatives are rarely employed in favor of synthetic ones [22]. Although synthetic preservatives are far more cost-efficient and highly effective than natural ones, they pollute the environment and have mutagenesis effects on non-target organisms [23]. Food preservatives are seen as potentially harmful to consumers’ health. Many investigations have concluded that pharmacological testing as well as toxicological testing would be suitable [22]. Numerous studies have revealed that volatile oils derived from different plants have enormous health advantages for users [2]. Because essential oils are natural chemicals that have bactericidal, fungicidal, insecticidal, and therapeutic effects, as well as distinctive smells, they have been employed in the food, pharmaceutical, and cosmetic sectors [24]. Thus, using essential oils to preserve food is a natural substitute for chemical methods [25] due to historical applications as potent antimicrobial agents [19]. Studies have concluded that most of these volatile oils show antimicrobial action, especially against some bacteria [26]. The potential to use natural, safe, eco-friendly, affordable, renewable, and easily biodegradable antimicrobial chemicals for food preservation in the near future is thus made possible by the many qualities of essential oils [3]. Table 2 shows the main chemical compounds of essential oils that provide antimicrobial activity.

In terms of food rotting, some of the most hazardous and well-known fungus species are found in the genera *Aspergillus* and *Penicillium*. There are species of *Chaetomium* and *Trichoderma* that also create mycotoxins that are detrimental to food losses and harmful to the health of humans and animals [31]. Studies conducted in vitro have demonstrated the antibacterial capabilities of essential oils against yeasts, molds, and Gram-positive and Gram-negative bacteria. *Bacillus cereus*, *Salmonella typhimurium*, *Escherichia coli*, *Listeria monocytogenes*, and *Staphylococcus aureus* are some of the microorganisms that have been investigated the most [32]. By hydrodistilling coriander leaves, Matasyoh et al. (2009) extracted their essential oil and assessed its antibacterial activity in vitro. Aldehydes and alcohols made up the majority of the oil, comprising 56.1% and 46.3% of the mixture, respectively. The extracted oil was tested for its ability to inhibit the growth of Candida albicans, a fungal pathogen, as well as Gram-positive (*Staphylococcus aureus*, *Bacillus* spp.) and Gram-negative (*Escherichia coli*, *Salmonella typhi*, *Klebsiella pneumonia*, *Proteus mirabilis*, and *Pseudomonas aeruginosae*) bacteria. The essential oil had a considerable effect on the other bacteria examined, but *P. aeruginosae* was the only one to exhibit resistance [33]. Twelve essential oils were investigated by Rattanachaikunsopon and Phumkhachorn (2010) for their potential as antimicrobials against various strains of *Campylobacter jejuni*, a bacterium that causes foodborne illnesses all over the world. The authors demonstrated that when tested against all strains, the essential oil had the strongest antibacterial efficacy [34]. Naturally occurring antibacterial and flavorful compounds, essential oils of cinnamon, mustard, thyme, and oregano are usually considered safe [32]. Furthermore, recent research has demonstrated the antibacterial action of essential oils of various plants against bacteria, yeasts, and molds, including garlic, basil, coriander, citrus peel, eucalyptus, ginger, rosemary, and peppermint. The antibacterial activity of essential oils, the amount of their primary constituents (terpenoids, phenols, and aldehyde compounds), and the kinds of microorganisms they can inhibit or inactivate are all related to how hydrophilic or hydrophobic they are [28]. The process of trapping solid, liquid, or gaseous components in matrices (encapsulants) that can support and eventually release their contents under particular circumstances is known as encapsulation [35]. Capturing viable and functional cells within a semi-permeable matrix is the goal of cell encapsulation technology [36]. To prolong the shelf life of sensitive base materials and lessen their reactivity, it can be used to shield them from their external environment, such as heat, moisture, air, and light. Selecting an appropriate encapsulating agent is a crucial undertaking that relies on the attributes of the starting material and the final product’s features [37]. Another advantage of using encapsulation is that the protective coating of the capsules remains intact, and therefore the encapsulated material does not easily migrate through the product [35].

At standard temperature and pressure, volatile oils are extremely unstable and volatile. As a result, the application of volatile oils requires their processing into solid forms like capsules, films, or spheres; semi-liquid forms like gels or liposomes; and liquid forms like emulsions, micelles, and liquid solutions [38]. Gums, chitosan, maltodextrins, starches, and proteins are examples of coating materials that can be used to encapsulate volatile oils [39]. Proteins and polysaccharides are examples of biopolymers that can be utilized to make food-grade capsules [37]. Encapsulation can be achieved by different processes. Examples include emulsification, molecular complexation/inclusion, antisolvent precipitation, the solvent emulsification–evaporation technique, the supercritical fluid technique, spray drying, electrospraying, and lyophilisation [35].

This paper aims to provide an overview of the current advances in volatile oil encapsulation technologies and their applications in the food industry. A variety of natural polymers utilized in the food industry for encapsulation are reviewed, as well as new research on food products supplemented with encapsulated volatile oils. Also, a distinct section is provided to highlight the current advances in dairy products enriched with encapsulated volatile oils.

## 2. Natural Polymers Used for Encapsulation

**Figure 1:**
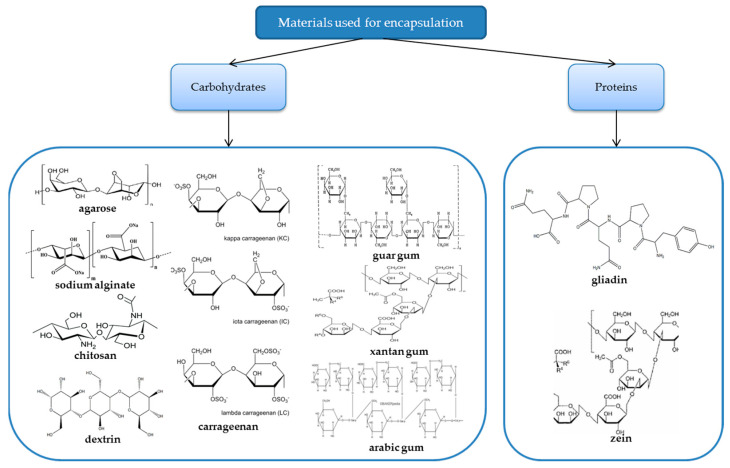
Chemical structure of the main materials used for encapsulation in food applications.

Materials for food applications that are utilized for encapsulation must be stable in food systems during processing, storage, and consumption, and they must also be suitable for human consumption and have good biodegradability [35]. The main advantages of using natural polymers for the encapsulation of volatile oils are the following: the selected materials are for food use, they do not affect the properties of the oils, having a fairly resistant and flexible structure from a mechanical point of view, and they ensure a gradual release of the oil from the inside, having an ideal porosity without modifying the properties of the encapsulated agent. As the encapsulation of volatile oils is a practice in its infancy, a major drawback would be the high cost that it can generate for the finished product. Also, the development of laboratory technology may lead to the formation of very thin films using these polymers. Figure 1 shows the chemical structures of the main materials used for encapsulation in food applications.

### 2.1. Bibliographic Research Methodology

The searches were performed in the Clarivate Web of Science database. The search terms were as follows: “encapsulation” AND “volatile” AND “oils” AND “agarose” AND “carrageenan” AND “alginate” AND “chitosan” AND “dextrin” AND “gum” AND “gliadin” AND “zein”. First, the abstracts and titles were reviewed; studies that lacked details about the natural polymers utilized in encapsulation were disqualified. After sorting, the chosen articles were thoroughly read.

### 2.2. Agarose

The polymer agarose [40] is obtained from the cell walls of red algae (*Rhodophyceae*) [41], which includes *Gracilaria* and *Gelidium*. Agarose’s primary structural components are 3,6-anhydro-α-L-galactopyranose and β-D-galactopyranose, which alternate [36]. Agarose beads exhibit excellent porosity, mechanical resilience, inertness to chemicals and physical stimulants, and high hydrophilicity [36]. Agarose is a gelling agent with a wide range of uses, including in the biomedical industry, where it is employed in the synthesis of biomaterials for the treatment of diabetes and the regeneration of the neurological, cardiac, bone, and corneal systems [42]. The solid–gel transition temperature and gel strength of several commercially available agarose varieties vary. Some of them have a solid–gel transition at about 37 °C, which makes them suitable for cell encapsulation [36]. Agarose has a large and unexplored potential for the encapsulation of probiotics, given its ability to block the penetration of oxygen into liquid media, and it is used to grow anaerobic bacteria [41].

### 2.3. Carrageenan

Carrageenan is a water-soluble anionic polysaccharide that is extracted alkalinely from red algae (*Rhodophyceae*). Similar to agarose, it is a galactan made up of repeating β- and α-D-galactose sequences with different ratios of sulfate groups. β-galactose is D in carrageenan and L in agarose [36]. With one, two, and three sulfate ester groups, respectively, kappa (κ), iota (ι), and lambda (λ) are the three most significant forms of commercial carrageenan [43]. Carrageenan is most frequently utilized as a medication carrier, even though it naturally possesses anticancer and anticoagulant qualities [44,45].

A polysaccharide that was extracted from seaweed, κ-carrageenan is frequently utilized as a gelling agent in the food and pharmaceutical industries. When cationic cross-linking agents such as metal ions, peptides, and proteins are present, anionic κ-carrageenan molecules can form hydrogel matrices. As a result, the injection approach can be utilized to produce hydrogel beads [46]. κ-Carrageenan creates spherical composite microcapsules with a consistent shape that are held up by calcium ions and comprise a web of biopolymers [47].

While κ-carrageenan is the most often utilized type, it also creates larger molecular weight fragments after hydrolysis in the gastrointestinal tract and is generally less toxic when consumed [43]. Iota-carrageenan is a sulfate hydrocolloid that is widely used in pharmacological, medicinal, and non-food applications. It is derived from seaweed [48].

### 2.4. Alginate

In the food and pharmaceutical industries, alginate is a naturally occurring polymer that is both biocompatible and biodegradable [49]. Found in the cell walls of brown seaweed, it is a linear polysaccharide made up of beta-D-manuronic acid (M) and alpha-L-guluronic acid (G) residues [50]. High-G-content alginates have been demonstrated to have superior compatibility, making them ideal for applications involving cell encapsulation [51]. This type of gum dissolves in both warm and cold water, and when it reacts with acids or calcium salts, it creates permanent gels. Alginates’ characteristics differ depending on where they come from and are influenced by the molecular weight and M/G ratio [52]. Alginates’ remarkable gelling, stabilization, and rheological behavior, along with their great water-holding ability, have led to their employment in a wide range of applications. They are frequently utilized in mixtures with other natural and synthetic polymers to create films, beads, capsules, fibers, and blends [53,54].

Alginates are generally low-cost, biocompatible, low-damaging, and biodegradable gelling biopolymers. The kinetics of the cross-linking process and the molecular structure of the alginates utilized, despite their straightforward gelling procedure, have a significant impact on the quality of the gels that are produced [53]. Alginate-based materials’ chemical makeup, molecular weight, and gelling conditions can all be changed to customize their qualities for particular uses [50]. Alginate’s drawback is that it is hard to regulate the release of encapsulated material from the gel matrix due to its porosity, high permeability, and ease of degradation. Therefore, the easiest way to get around alginate’s drawbacks is to combine it with other biopolymers [49].

### 2.5. Chitosan

Chitosan is a polysaccharide that is frequently present in the cell walls of fungi as well as the exoskeletons of arthropods and crustaceans. Chitosan is an excellent material for creating films, gels, and microcapsules because of its inflexible chemical structure. Furthermore, its physical, chemical, and biological characteristics make it suitable for the creation of microcapsules holding active components [55]. Chitosan is a deacetylated version of chitin, a common polysaccharide found in crustacean shells, and it is a polymer of D-glucosamine and N-acetyl-D-glucosamine joined by beta (1–4) glycosidic linkages [56,57,58]. The Food and Drug Administration (FDA) [59] has classified this biopolymer as generally recognized as safe (GRAS) due to its natural, non-toxic, and inexpensive linear polysaccharide with a positive charge at low pH [60,61].

Chitosan has been widely employed as a matrix for encapsulating extracts, essences, and bioactive substances in a variety of forms [59]. Microcapsules coated with chitosan are used to shield the active components from environmental variables including pH changes and temperature changes. Chitosan can be used as a coating material to microencapsulate a variety of starting materials, including foods, oils, pigments, catalysts, and active medicinal components [55].

Most essential oils have a typical hydrophobicity, which has led to the development of a microscale and nanoscale encapsulation approach [57]. Although essential oils have antibacterial properties, their volatility prevents them from being employed extensively. Chitosan encapsulation is crucial for the essential oil’s gradual release, which prolongs the oil’s availability [55].

### 2.6. Dextrin

Dextrin is a modified starch that is often used in food applications and as an encapsulating agent for vegetable oils. It is produced by partially hydrolyzing starch [62]. These low-molecular-weight carbohydrates are created from the partial acid and/or enzymatic hydrolysis of starch or glycogen. As a result, they have less polymerization and display the branched α-(1→4)- and α-(1→4,6)-Glc structures of amylopectin and amylose, respectively [63]. Similar in structure to dextrin, maltodextrin typically consists of short molecular chains (2 < n < 20). The most prevalent forms of cyclodextrin are α-, β-, and ϒ-cyclodextrin, which have cyclic chemical structures and include six, seven, and eight dextrin units, respectively [35].

Dextrins are regarded as safe and reasonably priced raw materials (GRAS). They are extensively utilized in numerous applications, including adhesives, food, textiles, and cosmetics [63]. Dextrins are inexpensive, provide neutral flavor, and effectively shield flavor from oxidation when used as encapsulants. Carrot carotene and orange oil have been found to have longer shelf lives when hydrolyzed starch is used. Dextrin is often used in combination with other encapsulants such as gums and starch [35]. The unit of measurement for total reducing power, “dextrose equivalent” (DE), is used to express the degree of hydrolysis. Different structural properties can explain the differences in hygroscopicity, fermentability, viscosity, sweetness, stability, gelation, solubility, and bioavailability across dextrins with the same DE [63].

### 2.7. Gum

Natural gum, known as Arabic gum, is extracted from the sap of several kinds of acacia trees. It is a water-soluble complex mixture of polysaccharides and glycoproteins. It is edible, a well-known encapsulant that safeguards things that are encapsulated, and is employed as a stabilizer (E414) in the food business [35] due to its emulsifying properties and low viscosity. It is made up of a complex mixture of polysaccharides, glycoproteins, and arabinogalactan oligosaccharides [64]. Arabic gum is a natural source of fiber, carbohydrates (galactose and arabinose), and mineral salts (potassium, magnesium, and calcium). Because of its qualities, which include volatile retention, emulsification, low cost [65], negative charge properties, low viscosity, high solubility, convenience of use, inhibition of oxidation reactions, and colorlessness of solutions, this heteropolysaccharide is frequently employed as a coating material [66,67]. Syrups are mostly concentrated using it in the pharmaceutical industry [65]. Because of its exceptional functional qualities and ability to remain stable in a variety of harsh conditions, it is utilized as a stabilizer in the food industry [67]. In the agricultural sector, on the other hand, its primary application is the creation of bacterial microcapsules [65].

Guar beans are used to extract guar gum, also known as guaran, which is a galactomannan polysaccharide. It is edible and a natural substance (E412) [35]. This naturally occurring galactomannan polysaccharide is made up of α-D-galactopyranosyl units coupled by 1,6 bonds to a linear β-d-mannose chain linked by β-(1-4) linkages [68]. Guar gum’s capacity to establish hydrogen bonds with water molecules makes it useful for a wide range of industrial applications [69]. Additionally, it has an exceptional ability to thicken water [35], eight times more than other agents (such as corn starch or other gums), and just a tiny amount is needed to reach the desired viscosity. The food, paper, pharmaceutical, and cosmetic sectors all make extensive use of it [69].

Made from sucrose, cane molasses, and whey, xanthan gum is a polysaccharide biopolymer made up of glucose, mannose, and glucuronic acid [65]. It is utilized in food applications and possesses qualities similar to guar gum (E415) [35]. Numerous industries, including the chemicals, petroleum, cosmetics, food, and agriculture industries, employ xanthan gum. Additionally, several drinks, candies, frozen foods, and tinned goods include this gum [65].

### 2.8. Gliadin

Plant proteins like gliadin, which are sourced from wheat, are commonly employed in particle manufacturing and encapsulation processes. They are soluble in alcohol and water mixes but insoluble in water alone [35]. Unlike many other food proteins, these wheat-derived proteins are very hydrophobic, which makes it possible to encapsulate hydrophobic substances without the requirement for oily phases in the development of food-grade delivery systems [35,70]. Glycidin’s amphiphilic structure consists of two hydrophobic terminal domains that include several hydrophobic amino acids (phenylalanine, leucine, tryptophan, etc.) and a core hydrophilic domain that is rich in glutamine and proline [71]. According to recent research, gliadin can encapsulate sensitive functional compounds in food-grade nanoparticles created by the antisolvent technique. Furthermore, during digestion and gastrointestinal absorption, gliadin nanoparticles demonstrate exceptional mucoadhesive qualities, which is advantageous for raising the bioaccessibility of the integrated bioactive chemicals [71,72]. In 2015, Davidov-Pardo et al. used the straightforward antisolvent precipitation method to encapsulate resveratrol utilizing gliadin and hydrophilic pectin. The efficiency of encapsulation was enhanced by the utilization of hydrophilic biopolymers. The authors concluded that these particles represent a promising encapsulation technology for adding this nutraceutical ingredient to functional foods [35].

Solid gliadin nanoparticles have, however, also demonstrated several disadvantages, such as inadequate encapsulation and protection against charged bioactive substances [71]. The fact that some people have gluten intolerance and have consequently been recommended to remove all gluten protein from their diet is another drawback of employing gliadin [35].

### 2.9. Zein

A total of 30–60% of the total protein in maize is made up of zein, which is the primary storage protein [35,73]. Leucine, proline, alanine, phenylalanine, valine, and isoleucine are among the more than 50% non-polar amino acids it contains; basic amino acids and acids are absent [74]. It is soluble in an ethanol–water mixture but insoluble in pure water or ethanol. It can be readily transformed into microparticles and spherical nanoparticles using electrospinning or a straightforward solvent fighting method [35].

Zein has been effectively utilized to create biodegradable food packaging layers and to encapsulate a variety of bioactive substances. This plant protein’s non-toxicity, biodegradability, and biocompatibility have made it useful in adhesives and coatings for food, pharmaceutical, and biomedical applications. Prolamin is a hydrophobic protein with a strong oxygen barrier and heat stability characteristics. Although electrospun zein nanofibers are simple to make and have uniform, flexible architectures, their limited water stability and mechanical qualities make them unsuitable for many applications [35,75]. Zein is a common coating substance used to deliver drugs with a specific target. Its most significant characteristic is that hydrophobic components—like fatty acids, fish oils, and essential oils—can be readily contained in zein particles provided they dissolve in aqueous alcohol solutions alongside zein [73].

## 3. Latest Advances in Food and Dairy Products with Encapsulated Volatile Oils

### 3.1. Bibliographic Research Methodology

The use of volatile oils encapsulated in various materials has been increasing, as evidenced by the number of publications in recent years. The database searched was Web of Science, and a first search was generated using the words “encapsulated volatile oil food products”. This yielded 102 results, 84 of which were articles and 18 were review articles. For the current study, only articles from 2013 to the present were kept. The number of articles dealing with this topic increased from year to year, with only 1 article being published in 2013, reaching 15 articles in 2021, 18 articles in 2022, and 6 articles in 2023.

To highlight studies that included the addition of encapsulated volatile oils in dairy products, the following searches were performed in the same database: “encapsulated volatile oil dairy products”, resulting in 5 articles, and “encapsulated volatile oil milk”, resulting in 18 articles.

### 3.2. Food Products with Encapsulated Volatile Oils

Volatile oils are an excellent source of protection for foodstuffs against various micro-organisms and for maintaining the quality of meat products, dairy products, fruit, vegetables, or fish [76,77].

According to Fadel et al. (2019), cinnamon essential oil is effective. Therefore, by incorporating cinnamon essential oil into biscuits, it was discovered that the oil served a dual purpose as an antioxidant and for flavoring. When maltodextrin was used as a delivery solvent instead of propylene glycol, the essential oil was better protected [78]. Hossain et al. created and evaluated chitosan-based nanocomposite films containing peppermint, tea tree, oregano, and thyme essential oils in 2019. During food storage, these bioactive coatings with antifungal qualities have a great deal of potential to regulate fungal development [79]. Kokina et al. tested the impact of sodium-alginate-encapsulated essential oils on jelly beans. Because of their bioactive and flavoring qualities, essential oils derived from yarrow, juniper, fennel, marjoram, and dill were employed to create a food additive that shows promise for application in functional meals [38]. A nanoemulsion system containing enterocin Gr17 and the essential oil of cinnamaldehyde was created and characterized by Duan et al. in 2023 and was applied to the storage of liquid smoked salmon fillets. This nanoemulsion system has been shown to improve fish products’ microbiological, physicochemical, and sensory qualities, making it a promising biopreservative technique and a viable substitute for traditional methods [80].

### 3.3. Dairy Products with Encapsulated Volatile Oils

Tița et al. have conducted several studies aimed at using various types of encapsulated volatile oils to improve the sensory, physicochemical, and enzymatic properties of various dairy products. In 2020, they studied the effect of volatile oils of basil, mint, fennel, and lavender encapsulated in sodium alginate on cow’s milk yoghurt, thus obtaining a food product with natural antioxidant compounds suitable for preventing and reducing the effects caused by pandemic stress in the human body [81].

**Figure 2:**
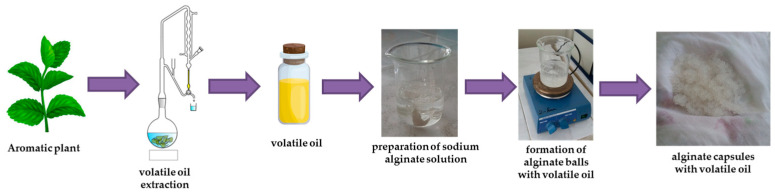
The process of obtaining and encapsulating volatile oil.

Interest in this topic was continued in 2022 and 2023, investigating the effect of peppermint, fennel, and lavender oil on cow’s milk kefir. Figure 2 shows the process of obtaining and encapsulating the volatile oil used by Tita et al. [82,83,84,85].

**Figure 3:**
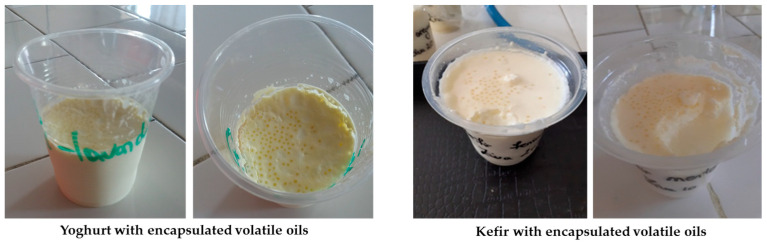
Dairy products with encapsulated volatile oil.

Sensory and textural properties [82], antioxidant capacity [83], the antimicrobial capacity of *Geotrichum candidum*, *Penicillium expansum*, *Aspergillus niger*, and *Escherichia coli*, and chemical composition using enzymatic methods [84] were highlighted. This resulted in an innovative dairy product with high nutritional value and health benefits for the consumer. Figure 3 shows samples of yoghurt and kefir with encapsulated volatile oil obtained by Tita et al. [82,83,84,85].

In 2018, Holgado et al. demonstrated how adding microencapsulated conjugated linoleic-acid-rich oil to skim milk components could protect volatile oils from oxidation. To maintain the chemical and physical stability of functional meals, free oil was provided as an alternative, and this resulted in a delayed oxidation process for the encapsulated oil component [85]. Yakubu et al. investigated adding microencapsulated microalgae oil to milkshakes in 2022. This kind of oil was found to enrich the content of n3 FAs, particularly DHA (concentration > 400 mg/g), which may have positive effects on cardiovascular health [86]. Terpou et al. investigated the use of Chios mastic gum as a matrix-forming substance and antibacterial agent to encapsulate probiotic cells to produce functional fermented milk. The results indicated that while mastic gum’s antibacterial qualities extended the shelf life of fermented dairy products, encapsulation increased *Lactobacillus casei* viability during chilled storage [87]. Another study conducted in 2020 investigated the effect of lavender extracts on two food products: ice cream and macaroons. The extracts were microencapsulated using various combinations of milk proteins and wall materials based on polysaccharides. Antioxidant activity was measured for ice cream that had microcapsules of lavender extract added, and the supplemented samples had increased antioxidant activity [88].

## 4. Conclusions and Future Perspectives

Volatile oils are characterized by the odor of the aromatic plants from which they originate and are end products of plant metabolism, accumulated and stored in various plant organs, cells, or secretory tissues. In recent years, the pharmaceutical and food industries have been using volatile oils because of their antimicrobial and antioxidant properties. Due to their sensitivity to environmental factors, volatile oils require special storage conditions. The use of encapsulation in different materials can be an alternative against the action of oxygen, heat, humidity, and pH. It is a process of packaging different materials in matrices that protect and release their contents under certain conditions. Depending on the method and materials used, encapsulation can be carried out in a variety of ways that result in the production of particles with various structures. The materials used for encapsulation in food applications must be of high quality, acceptable for human consumption, and stable during processing and storage.

This paper presented a series of natural polymers used for the encapsulation of various materials, especially volatile oils. The advantages and disadvantages of the use of these polymers were presented in scientific articles and research carried out in recent years. In addition to the presentation of natural polymers, the use of encapsulation of volatile oils in food products, in particular dairy products, was presented. According to the literature, there are not many studies highlighting the use of encapsulated volatile oils and their benefits in dairy products. The use of encapsulated volatile oils in dairy products is an excellent alternative for achieving a finished product with high nutritional value and offers the possibility of replacing synthetic preservatives. In this way, dairy products can be improved sensorily and physico-chemically, and the antimicrobial properties of oils can extend the shelf life of finished products. One of the primary drawbacks is that the industrial application of volatile oil encapsulation has not yet been fully explored. More research is required to include encapsulated volatile oils into as many food and dairy products as feasible, as well as to examine their physicochemical, sensory, microbiological, and antioxidant properties as well as their in vivo effects, before using them in an industrial setting. The use of the encapsulated version ensures that the characteristics of the oils are preserved for a longer period and that the active compounds are gradually released. In addition to being a great source of bioactive molecules that have many potential health benefits for consumers, volatile oils can also be studied and used as a viable substitute for artificial flavorings, preservatives, and antioxidants. In this sense, comparative studies are needed for the same products obtained with commercial additives and with encapsulated volatile oils.

## Figures and Tables

**Table 1:** Main chemical compounds of essential oils that provide antioxidant activity.

Plant Name	Scientific Name	Main Compounds	Reference
allspice	*Pimenta dioica* L.	eugenol, methyl eugenol, β-caryophyllene	[10]
caraway	*Carum carvi* L.	cumin aldehyde, γ-terpinene-7-al	[11]
cinnamon	*Cinnamomum zeylanicum*	(E)-cinnamaldehyde, benzaldehyde, (E)-cinnamyl acetate	[10]
cumin	*Cuminum cyminum* L.	trans-ocimene, cis-ocimene, γ-terpinene	[11]
ferulago	*Ferulago angulata* (Schlecht.) Boiss.	a-Pinen, z-β-ocimene, cis-β-ocimene	[11]
laurel	*Laurus nobilis* L.	1,8-cineole, α-terpinyl acetate, methyl eugenol, sabinene, eugenol	[10]
lavender	*Lavandula angustifolia* L.	camphor, eucalyptol	[10]
lemon grass	*Cymbopogon citratus*	geranial, neral, myrcene	[10]
mandarin	*Citrus reticulata*	L-limonene, ϒ-terpene, β-felandren	[10]
mint	*Mentha piperita* L.	menthol, mentofuran, 1s-neomethyl acetate, menthone	[11]
pomelo	*Citrus grandis* L.	limonene, α-terpinene, α-pinen	[10]
rosemary	*Rosmarinus officinalis* L.	1,8-cineole, camphor, α-pinen, limonene, camphene, linalool	[10]
sage	*Salvia officinalis* L.	β-Tujone, 1,8-cineole, camphor	[10,11]
yarrow	*Achillea millefolium* L.	limonene, a-pinen, borneol, thymol, carvacrol	[11]

**Table 2:** The main chemical compounds of essential oils provide antimicrobial activity.

Plant Name	Scientific Name	Used Part of the Plant	Antimicrobial Activity	Main Compounds	Reference
basil	*Ocimum basilicum*	whole plant	*C. albicans*, *S. aureus*	linalool	[18]
			*S. aureus*, *E. coli*, *C. albicans*	1,8-cineole, linalool, terpinen-4-ol, y-cadinol, T-cadinol, trans-α-bergamoten, eugenol, geraniol, germacren D	[27]
bergamot orange	*Citrus bergamia*	peel	*Campylobacter jejuni*, *E. coli*, *L. monocytogenes*, *B. cereus*, *S. aureus*	linalool, citral, linalyl acetate	[18]
black cumin	*Bunium persicum*	seed	*L. monocytogenes*, *Listeria grayi*, *Aspergillus flavu*	γ-terpinene, 1-felandrene, γ-terpene, couminaldehyde	[18]
chamomile	*Matricaria chamomilla*	fresh or dried flower heads	*Leishmania amazonensis*, *E. coli*, *P. aeruginosa*, *B. subtilis*, *S. aureus*, *S. pyogenes*, *Schizosaccharomyces pombe*, *C. albicans*, *Candida tropicalis*	α-bisabolol	[18]
cinnamon	*Cinnamomum zeylanicum*	peel	*Borrelia burgdorferi*, *E. coli.*, *S. aureus*, *P. aeruginosa*	carvacrol	[18]
			*M. smegmatis*, MRSA, *S. aureus*, *S. epidermidis*, *S. pyogenes*, *B. bronchiseptica*, *P. aeruginosa*, *K. Pneumoniae*, *C. albicans*	β-caryophyllene, trans-cinnamyl acetate, trans-cinnamaldehyde	[19]
			*Listeria monocytogenes*, *Escherichia coli*	cinnamaldehyde, caryophyllene, Ccryophyllene oxide, α-caryophyllene, γ-cadinene	[28]
clove	*Eugenia caryophyllata*	flower buds	*B. cereus*, *S. typhimurium*, *E. coli*	eugenol, β-caryophyllene	[18]
	*Syzygium aromaticum*	flower buds	*E. coli*, *S. aureus*, *S. typhi*, *P. aeruginosa*, *B. cereus*, *L. monocytogenes*	eugenol, eugenyl acetate	[18]
			MRSA, *S. aureus*, *P. aeruginosa*, *M. smegmatis*, *S. pyogenes*, *B. bronchiseptica*, *K. pneumoniae*, *C. albicans*	β-caryophyllene, eugenol, eugenyl acetate	[19]
coriander	*Coriandrum sativum* L.	seed	*B. subtilis*, *C. albicans*, *E. faecalis*, *E. aerogenes*, *E. durans*, *E. faecium*, *E. coli*, *K. Pneumonia*, *L. monocytogenes*, *L. innocua*, *P. aeruginosa*, *P. fluorescens*, *S. enteritidis*, *S. infantis*, *S. kentucky*, *S. typhimurium*, *S. aureus*, *S. epidermidis*	linalool, cis-ocimen, neryl acetate, y-terpinene	[29]
cumin	*Cuminum cyminum* L.	seed	*Staphylococcus aureus*, *Klebsiella pneumoniae*, *Salmonella typhimurium*, *Pseudomonas aeruginosa*, *Salmonella enteritidis*, *Escherichia coli*	a-thujene, α-pinen, sabinene, β-pinenene, β-mircene, α-phellandren, ∆-3-carene, α-terpinene, p-cymen, limonene, 1,8-cineole, β-phellandrene, γ-terpinene, α-terpinolen, terpinen-4-ol, α-terpineol, cumin aldehyde, safranal, cumin alcohol, β-caryophyllene, cis-β-farnesene, germacren-D, viridiflorol	[30]
fennel	*Foeniculum vulgare*	seed, leaves	*S. aureus*, *E. coli*, *A. flavu*	anethole	[18]
			*M. smegmatis*, MRSA, *S. aureus*, *S. epidermidis*, *S. pyogenes*, *P. aeruginosa*, *B. bronchiseptica*, *K. Pneumoniae*,	limonen, α-pinen, trans-anethol	[19]
lavender	*Lavandula angustifolia* L.	aerial part	MRSA, *S. aureus*, *E. coli*	linalool, borneol, camphor	[18]
			*M. smegmatis*, MRSA, *S. aureus*, *S. epidermidis*, *S. pyogenes*, *B. bronchiseptica*, *K. Pneumoniae*, *C. albicans*	lavandulyl acetate, linalyl acetate, linalool	[19]
lemon	*Citrus limon*	peel	*M. smegmatis*, MRSA, *S. aureus*, *S. epidermidis*, *S. pyogenes*, *B. bronchiseptica*, *P. aeruginosa*, *K. Pneumoniae*, *C. albicans*	γ-terpinen, β-pinen, limonen	[19]
lemon grass	*Cymbopogon citratus*	leaves	HSV-1, HSV-2, *S. aureus*, *E. coli*, *Gaeumannomyces graminis*	citral	[18]
			*M. smegmatis*, MRSA, *S. aureus*, *S. epidermidis*, *S. pyogenes*, *B. bronchiseptica*, *K. Pneumoniae*, *C. albicans*	geraniol, neral, geranial	[19]
mandarin	*Citrus reticulate*	peel	*S. aureus*, *E. coli*, *Penicillium italicum*, *Penicillium digitatum*	limonen, γ-terpinen	[18]
mint	*Mentha piperita* L.	leaves	*C. albicans*, *C. tropicalis*, *Pichia anomala*, *Saccharomycescerevisiae*	menthol, menthone	[18]
			*M. smegmatis*, MRSA, *S. aureus*, *S. epidermidis*, *S. pyogenes*, *B. bronchiseptica*, *K. Pneumoniae*, *C. albicans*	menthol acetate, menthone, menthol	[19]
			*Staphylococcus aureus*, *Klebsiella pneumoniae*, *Salmonella typhimurium*, *Pseudomonas aeruginosa*, *Salmonella enteritidis*, *Escherichia coli*	a-thujene, α-pinene, sabinene, β-pinene, β-myrcene, α-terpinene, p-cymene, limonene, 1,8-cineole, cis-β-ocymene, γ-terpinene, cis-sabinene hydrate, α-terpinolen, linalool, menthone, izomentone, mentofuran, menthol, terpinen-4-ol, neo-menthol, α-terpineol, pulegone, piperiton, mentil acetate, β-borbonene, β-caryophyllene, trans-β-farnesen, germacrene-D, viridiflorol	[30]
oregano	*Origanum vulgare*	leaves	*Trichophyton tonsurans*, *Trichophyton violaceum*, *Trichophyton floccosum*, *T. mentagrophytes*	carvacrol, timol	[18]
			*S. aureus*, *E. coli*, *C. albicans*	α-pinen, linalool, p-cymene, α-terpinene, γ-terpinene, α-tujen, β-caryophyllene, thymol, carvacrol	[27]
			*M. smegmatis*, MRSA, *S. aureus*, *S. epidermidis*, *S. pyogenes*, *B. bronchiseptica*, *P. aeruginosa*, *K. Pneumoniae*, *C. albicans*	carvacrol	[19]
			*Listeria monocytogenes*, *Escherichia coli*	β-cymen, linalool, carvacrol, γ-terpinene, caryophyllene	[28]
pomegranate	*Punica granatum*	seed	*S. epidermidis*	punicalagin, punicalin	[18]
rosemary	*Rosmarinus officinalis* L.	leaves	*C. albicans*, *C. tropicalis*	1,8-cineole, camphor	[18]
			*M. smegmatis*, MRSA, *S. aureus*, *S. epidermidis*, *S. pyogenes*, *B. bronchiseptica*, *P. aeruginosa*, *K. Pneumoniae*, *C. albicans*	α-pinen, camphor, 1,8-cineole	[19]
			*Listeria monocytogenes*, *Escherichia coli*	1R-α-pinen, eucalyptol, camphor, camphene, β-pinen, α-terpineol, bornyl acetate, caryophyllene	[28]
sage	*Salvia officinalis* L.	aerial part	*Staphylococcus aureus*, *Klebsiella pneumoniae*, *Salmonella typhimurium*, *Pseudomonas aeruginosa*, *Salmonella enteritidis*, *Escherichia coli*	cis-salvene, trans-salvene, tricyclene, a-thujene, α-pinene, camphene, sabinene, β-pinene, 1-octan-3-ol, p-myrcen, 3-octanol, α-phellandrene, α-terpinene, p-cymen, limonene, 1,8-cineole, cis-β-ocymene, γ-terpinene, cis-sabinene, α-terpinolen hydrate, linalool, α-thujonene, β-thujonene, trans-verbenol, camphor, borneol, δ-terpineol, α-terpineol, trans-sabinyl acetate, α-terpinyl acetate, β-caryophyllene, α-humulene, caryophyllene oxide, viridiflorol, β-selenene, humulene epoxide II	[30]
savory	*Satureja hortensis*	leaves	*S. aureus*, *Corynebacterium glutamicum*, *P. aeruginosa and E. coli*, *C. albicans*	carvacrol, timol	[18]
			*M. smegmatis*, MRSA, *S. aureus*, *S. epidermidis*, *S. pyogenes*, *B. bronchiseptica*, *P. aeruginosa*, *K. Pneumoniae*, *C. albicans*	linalool, para-cimen, thymol, γ-terpinene	[19]
ylang-ylang	*Cananga odorata*	flower	Hepatitis B virus (HBV), *Bacillus. subtilis*, *E. coli*, *S. typhi*, *Shigella shiga*, *Streptococcus-β- haemolyticus*, *A. flavu*	linalool, β-caryophyllene	[18]
			MRSA, *M. smegmatis*, *S. epidermidis*, *S. pyogenes*, *B. bronchiseptica*, *K. Pneumoniae*	linalool, geranyl acetate, trans-α-Farsen, β-caryophyllene, germacrene D	[19]

## Data Availability

Not applicable.
